# Ambient Intelligence Systems for Personalized Sport Training

**DOI:** 10.3390/s100302359

**Published:** 2010-03-22

**Authors:** Javier Vales-Alonso, Pablo López-Matencio, Francisco J. Gonzalez-Castaño, Honorio Navarro-Hellín, Pedro J. Baños-Guirao, Francisco J. Pérez-Martínez, Rafael P. Martínez-Álvarez, Daniel González-Jiménez, Felipe Gil-Castiñeira, Richard Duro-Fernández

**Affiliations:** 1 Universidad Politécnica de Cartagena, Campus Muralla del Mar, Antiguo Cuartel de Antigones, Cartagena, Spain; E-Mails: pablo.lopez@upct.es (P.L.-M.); hono.navarro@upct.es (H.N.-H.); pjguirao@upct.es (P.J.B.-G.); fjpmartinez@upct.es (F.J.P.-M.); 2 Universidad de Vigo, Campus Lagoas-Marcosende, Vigo, Spain; E-Mails: javier@det.uvigo.es (F.J.G.-C.); xil@det.uvigo.es (F.G.-C.); 3 Gradiant, Campus Lagoas-Marcosende, Vigo, Spain; E-Mails: rmartinez@gradiant.org (R.P.M.-A.); dgonzalez@gradiant.org (D.G.-J.); 4 Universidad de A Coruña, Rúa Maestranza, A Coruña, Spain; E-Mail: richard@udc.es

**Keywords:** ambient intelligence, contextual services, wireless sensor networks, sport training, machine learning

## Abstract

Several research programs are tackling the use of Wireless Sensor Networks (WSN) at specific fields, such as e-Health, e-Inclusion or e-Sport. This is the case of the project “Ambient Intelligence Systems Support for Athletes with Specific Profiles”, which intends to assist athletes in their training. In this paper, the main developments and outcomes from this project are described. The architecture of the system comprises a WSN deployed in the training area which provides communication with athletes’ mobile equipments, performs location tasks, and harvests environmental data (wind speed, temperature, *etc*.). Athletes are equipped with a monitoring unit which obtains data from their training (pulse, speed, *etc*.). Besides, a decision engine combines these real-time data together with static information about the training field, and from the athlete, to direct athletes’ training to fulfill some specific goal. A prototype is presented in this work for a cross country running scenario, where the objective is to maintain the heart rate (HR) of the runner in a target range. For each track, the environmental conditions (temperature of the next track), the current athlete condition (HR), and the intrinsic difficulty of the track (slopes) influence the performance of the athlete. The decision engine, implemented by means of (*m, s*)-splines interpolation, estimates the future HR and selects the best track in each fork of the circuit. This method achieves a success ratio in the order of 80%. Indeed, results demonstrate that if environmental information is not take into account to derive training orders, the success ratio is reduced notably.

## 1. Introduction

Wireless sensor networks (WSNs) link information technology with the physical world. Currently, WSNs are used in a broad range of applications: industrial process control, ecosystem monitoring or health monitoring among others. Nevertheless, the application areas are constantly growing. It has been stated that WSNs will provide intelligence to everything around us [1]. As sensor devices shrink, and their computing and communication capabilities increase, WSNs will get seamlessly integrated into the physical world (they are transparent to the users, in the sense that the users only perceive the output of the interfaces).

WSNs may be sensitive and responsive to the presence of people. Depending on the sensed data (e.g., biometrics, weather conditions, *etc*.), network devices must situate the user in a *context*, enabling applications to react to the changing environments. However, there are still pending research challenges, regarding hardware requirements, service accessibility, decision-making intelligence, human interfaces, system architectures, security, reliability, *etc*.

To some extent, the emergence of these intelligent systems is simply a natural evolution of the historical trend in computing and communications technologies, which are already of use in different fields. As an example, advanced sports training is often assisted by more or less complex computing devices carried by the athletes, which provide useful telemetry about their biometrics and practice related events. Among other parameters, they can monitor heart rate, track routes, measure speeds and distances, and so forth [2]. All these data are collected while training and recorded in a wrist or arm unit, to be analyzed by an external software, after each training session. This approach seems static in some sense, because the external software does not adapt itself to the athletes progress, nor it provides any advice while training.

**Figure 1.**
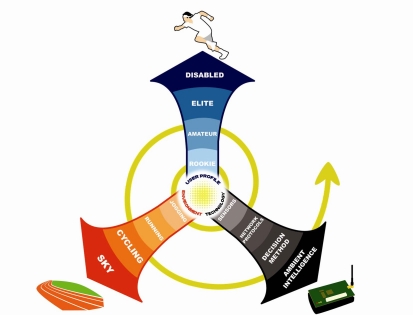
General vision of ambient intelligence aimed at Sports.

Supported by the Spanish Ministry of Education, Culture and Sports [3], project *Ambient intelligence systems to assist athletes with specific profiles*, pursues to provide personalized assistance to athletes in their training. As shown in Figure 1, there are three driving aspects in this project: athletes profile, environment, and computing and communications/decision technologies. Technology will provide adaptive coordination between the user and the environment, adapting the behavior of the system as it responds to changes in the environment or the user conditions, such as weather or athletes location. Since training conditions can rapidly vary, the system will be expected to take real time decisions to meet the user needs at their full potential. Thus, a decision engine, as well as efficient communications and localization protocols, are two key working areas in the project. This article presents its main advances so far.

The architecture proposed has been validated with a prototype for a cross country running case study. In this example, the goal is selecting suitable tracks for the athlete in order to maintain his/her heart rate (HR) within a defined target range, as the exercise endurance or the environmental conditions changes. Decisions depend both on user and on environmental conditions, as well as on information about the previous training sessions of the athlete. With these data the decision engine estimates the expected HR for the *future* track forks and informs the runner which one is appropriate to maintain the target HR.

The relationship between HR and sport activity is affected by many factors, such as temperature, age, sex, or mental stress, among others [4–7]. A simple and widely used strategy to control HR is to give acoustic feedback as soon as the target range is exceed to suggest user to reduce the speed. In this sense, our proposal *complements* this training feedback, providing more advanced control, since slope of the track and temperature also affect HR. We demonstrate that including this information affects notably to the quality of the estimation (see Section 6). Besides, in our proposal the focus lies on inferring the *future* HR for the different tracks, and selecting some that is suitable, instead of correcting HR when it is over range.

Summarizing, the main contributions of this work are:

- To design and demonstrate the feasibility of a communication/location architecture.
- To develop a dynamic decision engine based on machine learning tools which combines different data sources to improve decisions about training.
- To show the usefulness of the ambient intelligence paradigm in the area of sports.
- To test the system in a real outdoor training environment.

The rest of the paper is organized as follows: Section 2 discusses related work. Section 3 presents the system architecture, its components and the communication protocols. Sections 4 and 5 describe a system prototype and the methodology used for the implementation of the decision engine, respectively. The cross country running case of study is analyzed in Section 6. Finally, Section 7 concludes the paper.

## 2. Related Work

As described in [8], the use of chronometers, photocells, contact platforms, microphones, photo or video cameras, magnetic resonance and X-rays machines, movement sensors and, in summary, every sensing device for physical or chemical parameters, is nowadays common in athletes’ training. Many of these devices are not exclusive for elite athletes, but available to the general public (e.g., heart rate monitors). They are used for training monitoring, by athletes themselves or by their coaches, to improve physical performance.

The availability of these devices was the first step towards contextual services. Now, sport monitoring systems introduced real-time data collection, as well as user localization. Communications capabilities are provided by standard wireless network protocols (IEEE 802.11, Bluetooth/Wibree, Zigbee, *etc*.). Either these networks or specific ones (UWB, RFID, GPS, *etc*.) may support location [9]. Current developments aim at expanding the range of monitored data and providing useful actions and information based on them. WSNs [10] are one of the enabling technologies for that evolution.

In a first group, several developments of advance sensing devices in the field of sports can be identified. The focus of these works is on collecting data related to specific applications. However, these data are not used directly to obtain real-time feedback, but they are externally analyzed by human experts. Next, we describe works belonging to this group. A system to supervise elite sports training is proposed in [11]. Their work shows how sensors expand available data to study exercise execution. Three use cases are considered: table tennis, biathlon and rowing. In the case of table tennis, the system detects impacts of the ball on the table, computing its position by means of vibration triangulation, to display throw accuracy. In the biathlon case, a laser positioning system analyzes the motion of the rifle barrel before and after the shot. Finally, in the rowing case, the system calculates the effort that is applied to the oar. MarathonNet [12] is a WSN which monitors runners in marathon events. Sensors on runners collect data about HR, time and location. These data are sent via base stations along the trac to a central database, where they are analyzed *off-line*. Communication can be implemented by means of GPRS, WLAN, or a wired network link. The system in [13] assists professional skiers. Using accelerometers and force-sensing resistors, skiers can obtain data about their movements and visualize them, along with video footage, once the exercise has finished. SESAME [14] proposes the combination of two data sources, video and sensed parameters, to improve the performance of novice and elite athletes. Major competitions, like the Olympic games, typically pioneer the adoption of new technologies. As an example, in [15], the author introduces the SensorHogu systems, which embeds piezoelectric force sensors on body protectors, to recognize valid scoring kicks in Taekwondo. The commercial product Team^2^Pro [16] allows to record and study fitness data in real time for up to 28 players.

None of the previous works consider real-time feed-back. In other words, the human expert (e.g., the coach) carries this task. Some systems aim at simplifying this manual task are presented in [7,17,18]. They describe a system which monitors dynamic data from cyclists and their bicycles and provides *automatic* real-time feedback. For instance, the system can advise the group to change the formation, split, or to increase or decrease the speed. In [17] decisions consist of simple rules based on thresholds on monitored variables, whereas in [7,18] emphasis is put on the control algorithm taking into account the HR of the cyclist, the headwind, and other parameters. Reference [19] introduces a sensor system which provides immediate feedback to alert users about incorrect movements and body positions in snowboarding. The prototype uses sensors attached to the human body and inserted into the boots. The system detects body position (e.g., knee bending) and calculates deviations from positions previously captured from experienced subjects. However, the system is unaware of the surrounding environment (e.g., slopes) to improve decisions. In [20] a score system for golf swing motion is developed. The golfer body motion is captured by a wireless network of inertial sensors which extracts snapshots of the orientation data of the user body in the golf swing. The orientation information is compared with correct motion rules, and a score of the exercise is computed. Reference [21] also develops a feedback system based on sensors which capture the movements of a golf swing. The swing motion is preprocessed locally and sent to a base station (e.g., a PDA) for further analysis. This system does not consider environment conditions and, thus, does not incorporate devices embedded in the environment. The quality of the swing motion is expressed as the amount of deviations from the target line, and is computed by linear discriminant analysis.

As the last works described our proposal intends to provide *real-time feedback* to the athletes. Previous works use information of the athletes or in some cases from their close environment. We expand the environmental characterization to include data from the whole training field. Including this information improves the quality of decisions in some sports (e.g., running) as we demonstrate in Section 6. In addition, our inference system is aimed at estimating *future* athlete performance, rather than only determine the current quality of the exercise. Therefore, feedback assists athletes in order to maintain their target activity, instead of modifying it when it is not correct. Another specific characteristic of our prototype is that, during the training, it evaluates if the decisions were correct and incorporates this information to improve further feedback.

## 3. System Architecture

Our goal was designing a general architecture to support athlete and environmental monitoring, in order to provide real-time feedback for training improvement. The infrastructure is mainly oriented towards open-field sports, such as running, cycling or skying. In these scenarios, athlete performance depends not only on physical conditions, but also on terrain conditions (slope, temperature, wind, *etc*.). Environmental sensing is less critical for indoor sports and athlete sensing becomes highly specialized, as described in Section 2. Therefore, our approach focus on outdoor scenarios.

Two types of data sets are important to characterize user performance:

- A *static* set, which has information that does not change along training, from the environment (e.g., terrain slopes, intrinsic exercise, pathway configuration, sensing nodes position, *etc*.) and from the user (e.g., performance profile, age, skill, training goals, *etc*.).
- A *real-time* set, with updated environment information (temperature, wind, visibility, etc.) and athletes’ data (heart rate, body movement, elapsed training time, *etc*.).

A control element processes these data and issues commands to direct user exercise. Two approaches are possible for the implementation:

- A user-centric implementation, where the user equipment has the sensing devices necessary to monitoring data from the environment and from the athlete. This terminal is also in charge of taking the decisions to assist the athlete.
- A distributed approach, where different elements carry out different tasks. A WSN performs environment monitoring, whereas the user equipment monitors the athlete. Data processing is performed either by some external element or by a node of the WSN.

It can be argued that commercial smart-phones may be used in a user-centric implementation, due to the their high computational capabilities (in fact, the method that we propose for the decision engine requires a very low computational power, see Section 5). The major drawback of smart-phones is their scarce sensing possibilities. Usually, they do not have sensors to monitor environment (temperature, humidity, *etc*.). Moreover, some environmental data are difficult to collect accurately by a mobile terminal (e.g., wind speed). However, some specific user terminal can be developed (as the one used in this project). In this case, particular sensor devices can be included to monitor the environmental data and the athlete. Though this approach is possible, only the data from the current position of the athlete is available, therefore limiting the effectiveness of the assistant. A solution could consist of using the last available data for a position, if the environmental conditions change slowly (e.g., temperature). But there will be uncertainty in the places not visited by the athlete. If more than one athlete is training it is also possible to share environmental data among users, transmitting these data to a server via a cellular or wireless link connection.

The distributed approach combines static sensors with mobile ones, thus, enriching the data available to take decisions. Nevertheless, the cost of deploying a WSN must be considered. The overall costs of the equipments for the prototype deployed in the case study (see Section 6) was around 5,000. In a permanent installation, for example in a stadium, this cost is negligible in comparison with the cost of the buildings. For a temporary one, a clear requirement in the design is that the nodes auto-configure themselves for any arbitrary topology, to minimize installation costs. As an estimation for the user-centric approach, the cost of the user terminal developed in this project is around 1,000.

In this work we have selected the distributed approach. The reason is twofold. First, the goal of this work is focused on developing a suitable decision engine, rather than on developing advanced user equipments. Second, the quality of the data from the environment may affect the operation of the decision engine. In this case the WSN provides accurate data to test our proposal.

The rest of this section describes the architecture and protocols of the system.

### 3.1. System components

**Figure 2.**
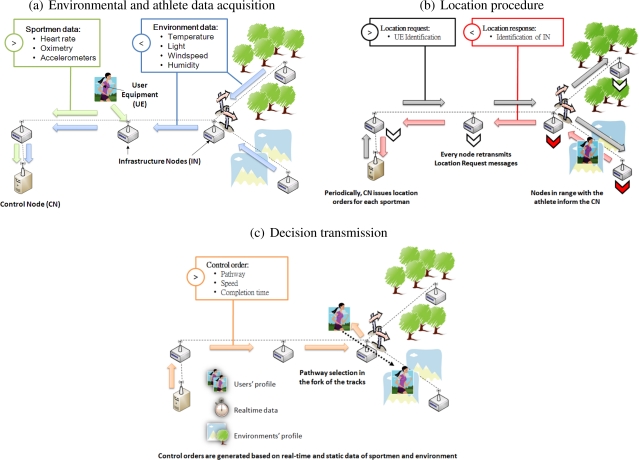
Data flow of the sensed data, the localization information, and the commands from the CN.

Figure 2a shows a typical system deployment.

It consists of the following elements:

- Infrastructure Nodes (IN), which cover the training area. These nodes sense environmental variables and relay data to/from the User Equipment or other INs to the Control Node. Communication relies on a wireless link. In addition, IN nodes also serve as an auxiliary network to perform athletes location in the training area. An IN is composed by a processing unit, a wireless interface including directional communication antennas, and a power supply. Directional antennas increase the communication range between IN nodes, so that less nodes are necessary, thus reducing network installation costs.
- User equipment (UE). This is the device carried by the user, which includes a wireless transceiver to communicate with the IN, and sensing elements to monitor athlete physical parameters. The UE also includes one or more user interfaces (acoustic, visual, *etc*.) to deliver training orders. The UE has the same functional blocks as an IN, but, as its weight and volume are constrained, internal antennas must be used, with a low range since they are less directional. However, precise aiming is not required for low directional antennas, and thus the UE is less obtrusive for the physical activity.
- Control Node (CN). It can be considered as a particular IN, which collects data from all IN nodes and the UE, and analyzes them to assist user training. CN processing requires access to the static information set, which is stored in databases or standard files.

### 3.2. Network deployment and communication protocols

The scenario described in the previous section is a WSN with fixed sensing elements (IN) and mobile ones (UE). As in a typical WSN, energy constraints are demanding for these elements, since they usually run unattended. Thus, power saving is a mandatory requisite of protocol design. In order to reduce the burden of protocol signaling, it is assumed that the IN nodes are arranged in a tree topology (data flows from the CN to the INs, and *vice versa*, suggesting also a topology of this type with the CN as the root). This topology simplifies routing task and can be easily computed at network startup (and updated periodically). Several protocols have been proposed for this task (e.g., Level Discovery Protocol [22]). We have implemented a simple version of this protocol that updates topology every hour, using the CN as the coordinator and the root node of the tree.

Notice that communication ranges differ between IN-IN and IN-UE links, since UE antennas are less directional than IN ones, creating *shadow areas*, where UE can not communicate with the WSN. This effect has been taken into account in the design of data communication and location protocols, which are described next.

The Medium Access Control (MAC) protocol used is a variation of B-MAC [23]. B-MAC provides unicast and broadcast transmissions. B-MAC is focused on energy consumption minimization, which is mandatory in a real WSN deployment. Unicast transmissions include an Automatic Repeat reQuest (ARQ) mechanism that guarantees packet delivery in case of wrong packet transmissions. Notice that transmissions between INs and UEs in shadow areas are automatically corrected by ARQ.

The position of the INs must be selected to guarantee network connectivity. For network planning purposes, there exist some specialized tools, but they usually ignore practice-related considerations (for instance, if wind sensing is interesting, some INs in open ground positions are desirable). An optimization method to determine the best sites, while guaranteeing connectivity is described in [24].

#### Data exchange

**Figure 3.**
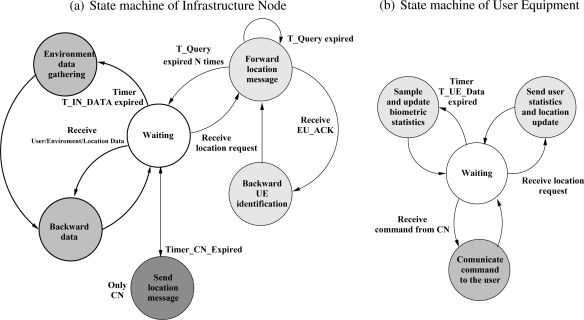
State machine diagrams.

Figure 3a,b shows the state diagram of the UE and the IN, respectively. Data is gathered by the INs and UEs periodically (every *T_IN_Data_* and *T_UE_Data_* seconds respectively). Once the data are collected, the INs send them to their parent nodes (see Figure 2a), which backward them again until they eventually reach the CN. To reduce communication cost, the UE processes data internally to compute their moving averages and standard deviations. The resulting packets with those statistics are delivered to the CN via the INs when the UE responds to a location update query.

The CN issues orders for specific UEs by means of command packets (see Figure 2c). These commands are delivered when the athlete must undertake some action, such as selecting another track. The CN delivers the order by transmitting the command to a specific IN, which, in turn, handles its transmission to the UE. The specific IN is selected according to the UE position and its movement direction. Both information are provided by the location procedure.

#### Location procedure

The system also determines athletes location. Figure 2b illustrates this process. It operates as follows: the CN periodically starts a search sending a location request message, including the identity of the nodes it looks for, or a special value indicating that all nodes must respond. Immediately, the INs forward this query to all their leaf nodes using broadcast packets. If one or more of the sought nodes receive this packet, they answer to the IN with a unicast packet, which also includes the last athlete statistics. Then, the IN backwards the packet to the CN. Each IN retransmits the broadcast query packet every *T_QUERY_* seconds for *N_QUERY_* times, since the broadcast packet can be lost if the athlete lies temporary in a shadow area (broadcast messages are not protected by ARQ). Finally, the CN receives the information of all the INs which detected the searched node. The athlete movement direction may also be computed by the CN from the previous location records.

## 4. Implementation

A prototype based on the architecture described in the previous section has been implemented using standard WSN hardware (MICAz and IMOTE2 motes from Crossbow Technology [25]). This prototype has been tested in a cross-country running application (described in the next section). Nevertheless, it can be easily adapted to other sport activities, such as cycling, walking, *etc*. In the following paragraphs the implementation of each component is described, as well as the parameter selection for the protocol.

### 4.1. IN implementation

Each IN requires sensing, processing, and communicating capabilities, as stated in Section 3. We selected the MICAz mote [26], based on the CC2420 chip [27], as the IN core. It works in the 2.4 GHz band, and it is compliant with the low power Zigbee/IEEE 802.15.4 [28] physical interface. MICAz software relies on the open source event-oriented TinyOS operating system [29], which is a reliable platform for ad-hoc protocol programming.

Environmental sensing is carried out by a MTS400 sensor board [30], designed for Crossbow motes, which measures light, temperature, humidity, and barometric pressure, although in our testbeds (Section 6) we only activated temperature sensing.

In addition, each MICAz is equipped with two panel antennas (a 2.4 GHz Stella Doradus 24-8080 planar antenna [31]) to increase communication range. The reason is twofold: (i) to cope with radio propagation issues (caused by natural obstacles like knolls, woodlands, *etc*.) which may worsen communication among INs; and (ii) to increase communication range between the stations, allowing a lower density of INs, and therefore, reducing installation and operation costs. In actual deployments, it was verified that communication range may reach up to 180 meters, although the INs were installed with a maximal distance of 150 meters in between, as a security margin and to increase the precision of the location algorithm. Notice that, with the default antennas that are embedded in the MICAz motes, the typical range is less than 100 meters.

**Figure 4.**
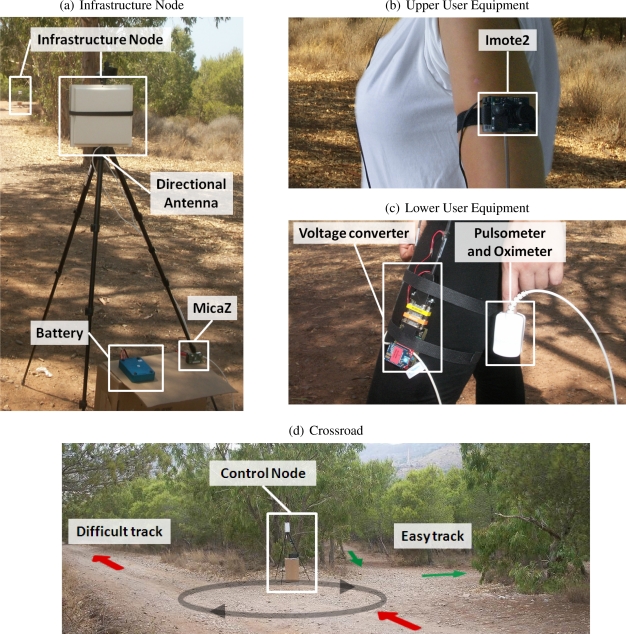
Deployed hardware.

Figure 4a shows a picture of two IN in a running track Figure 4d.

### 4.2. CN implementation

The CN can be considered a special IN, because it has the same sensing and data transmission capabilities. Moreover, the CN also implements the *intelligence* of the system: it issues commands to the network and to the users in order to fulfill training goals. Its decisions are transparent to the user, which only receives the training advice through his/her UE. In this implementation, the decision engine selects the best path to achieve a target HR. The classification procedure to compute the HR is based on a (*m, s*)-spline approximation. Section 5 describes this method in depth.

The location of the CN in our network deployment is shown in Figure 4a,d.

### 4.3. User equipment

As in the IN case, the UE combines sensing and communications functionalities. Nevertheless, the UE is designed to sense human biometrics, instead of environmental parameters. Human biometrics variables usually change faster than environmental ones. Therefore, sample frequencies must be higher. In addition, to avoid continuous packet transmission, these samples are processed in the UE, and only their statistics are transmitted. The UE also delivers training advices to the athlete. Thus, the UE is the interface between the system and the user. In the implementation, audio feedback was used. However, audio messages for the user are not actually transmitted over the WSN. Instead, the UE contains the different possible commands already stored in its flash memory (several hundreds of voice commands can be stored in this 32 MB memory). The CN only sends the identification of the message, and the UE reproduces this message. Audio feedback allows users to receive complex commands, not just binary orders. Since only the message identification is transmitted, the required bandwidth and energy to send the command is minimal.

To perform these operations, the UE is composed by several modules, as shown in Figure 4b,c. The main module is the Crossbow IMOTE2 IPR2400 [32], a wireless sensor network platform that can be expanded with extension boards to customize the system to a specific application. IMOTE2 also includes an 802.15.4 radio (CC2420) with a built-in 2.4 GHz antenna for IN-UE data communications. A IMB400 multimedia board [33] is used with the IMOTE2, it includes an audio output to play the speech messages. The IMB400 also has a CCD color camera and audio capture, which could be used in future system applications.

For human biometrics sensing, an integrated pulse oximetry device (iPOD model 3211, from Nonin Medical company [34]) was used. This sensing device takes measures of heart rhythm and oxygen level with a low power consumption. The iPOD was chosen due to its lightness, size, and easy integration in the UE (*via* a RS-232 interface). A specific driver in TinyOS for connectivity and iPOD control was also developed. One advantage of this device is that it automatically discards wrong samples of the HR, avoiding HR sample errors (actually, iPOD returns an error code if the HR was not sensed). In our experiments the rate of successful returned samples was usually higher than 90%, in some experiments (e.g., if the user temporarily moves the iPOD) it drops, but since HR is measured with a high sample frequency, enough data is always collected.

### 4.4. Protocol parameters selection

In order to tune protocol operation, different parameters and timers (introduced in Section 3) must be established. Some of them are related to the athlete activity (e.g., speed), whereas others are related to environmental change rate (the sampling periods) in order to limit message exchange to minimize the impact on energy consumption. The selection of parameters is discussed next. Table 1 summarizes the parameters for the specific example of Section 6.

- *T_QUERY_* is the elapsed time between IN transmissions of location update requests. It is related to the shadow time, *T_SHADOW_*, which an athlete may experience between consecutive INs. The criterion was *T_QUERY_* = *T_SHADOW_*, since this guarantees that, in the worst case, the number of lost packets is just one. Otherwise, for lower values, more packets may be transmitted (and lost) while the user is in the shadow area. For higher values, the delay of the communication between the IN and the UE will increase.
  To compute *T_SHADOW_* let us denote *v* as the average expected user speed, and *D_SHADOW_* as the length of the shadow area. Then,
  (1) $$T_{\mathrm{QUERY}}=T_{\mathrm{SHADOW}}=\frac{D_{\mathrm{SHADOW}}}{v}$$
  Besides, *D_SHADOW_* can be computed as a function of IN antenna directivity gain (*G_IN_*), the directivity gain of the UE (*G_UE_*), IN output power (*P_IN_*), UE output power (*P_UE_*), and the distance between INs (*D*).
  (2) $$D_{\mathrm{SHADOW}}=D\left(1-2\sqrt{\frac{G_{\mathrm{UE}}P_{\mathrm{UE}}}{G_{\mathrm{IN}}P_{\mathrm{IN}}}}\right)$$
  Hence,
  (3) $$T_{\mathrm{QUERY}}=\frac{D\left(1-2\sqrt{\frac{G_{\mathrm{UE}}P_{\mathrm{UE}}}{G_{\mathrm{IN}}P_{\mathrm{IN}}}}\right)}{v}$$
  For a reference training speed of 15 km/h (4.16 m/s) and since in our testbed *G_IN_* = 11.15 dB, *G_UE_* = 1.41 dB, *P_IN_* = 0 dBm, *P_UE_* = 0 dBm, and *D* = 150 meters, then *T_QUERY_* ≈ 13 s.
- *N_QUERY_* is the number of times that an IN tries to locate UEs nearby. It is configured to be the expected number of intervals of *T_QUERY_* s that a user stays in the IN range:
  (4) $$N_{\mathrm{QUERY}}=\frac{D}{v}\frac{1}{T_{\mathrm{QUERY}}}$$
  With the reference configuration, we select *N_QUERY_* = 3 (the actual value 2.77, but the parameter is an integer, so it is rounded up).
- *T_CN_* is the elapsed time between two location procedures initiated by the CN. The minimal interval corresponds to the expected time in IN range:
  (5) $$T_{\mathrm{CN}}=N_{\mathrm{QUERY}}T_{\mathrm{QUERY}}=\frac{D}{v}$$

Higher values reduce energy consumption, at the expense of a lower location accuracy.

With the reference configuration, the minimal value should be *T_CN_* ≈ 36 s.

## 5. Decision Engine: (*m, s*) Degree Splines Classification

The goal of our case study is to select suitable tracks in order to maintain the HR of the athlete in a target range. We assume that each track has its particular conditions of hardness and temperature. Thus, the decision engine must infer the expected HR at each possible track fork, and select one where the expected HR will be in the given range.

**Figure 5.**
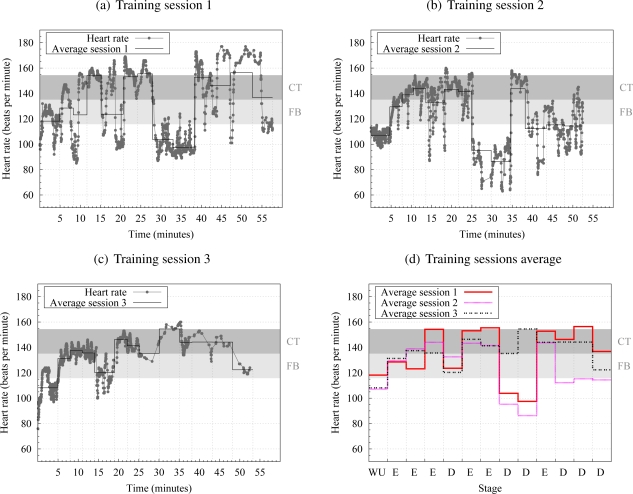
HR evolution in three training sessions.

The problem of evaluating the expected HR is complex. Analysis of HR dynamics by methods based on chaos theory and nonlinear system theory have received attention recently, due to observations that suggest that the mechanisms involved in cardiovascular regulation likely interact with each other nonlinearly [35–37]. Thus, characterizing HR and athlete performance is even more difficult. As an example of its difficulty, Figure 5 shows results for three training sessions, which were performed as part of the experimental validation in Section 6. It is interesting to observe in Figure 5d how the same part in different sessions produces so different average HR values. This is because athletes psychology and physical condition have an important role in performance. They can induce the athlete to increase physical activity, and, accordingly, HR in the easy section of the course; or the athlete to rest in the same section, leading to activity and HR decrease. For example, this is possibly the reason why session 3 ends several minutes before sessions 1 and 2 do. Note as well that HR increases are not directly related to hardness or temperature increases: e.g., the hardness increase from track 7 to track 8 (see Figure 5d) produces a sudden HR drop.

There exist several numerical and statistical methods to determine the relationships hidden in complex processes where many variables can be involved. These methods have been utilized to predict physiological parameters, such as HR, systolic blood pressure, or body temperature. Some possibilities are: Support Vector Machines (SVM) which have been successfully applied to medical decision support. Reference [38] describes how to mine medical knowledge from time series of high-dimensional numerical data describing patients in intensive care. A SVM is used to learn how and when a drug dose must change. Also, in [39] it is proposed to improve the diagnosis of tuberculosis infections by means of SVM image classification. Nevertheless, for sportsmen training purposes, a SVM statistical method requires a huge amount of time series data of many training sessions, in order to feed the knowledge base of each athlete appropriately. Therefore, the approach is impractical. Based on HR and 3D acceleration signals the strategy in [40] employs Feed-Forward Neural Networks (FFNN) to predict the next time step in the HR sequence. The predicted HR follows the variance of the real HR, although with noticeable differences in some cases. In [7] a dynamic heart rate prediction model is also used by a model predictive controller to optimize the cycling training. To sum up, many other techniques may be used in this problem, such as k-NN classifiers, fuzzy logic, *etc*.

We have formulate our problem using three different approaches: (i) using SVM classifiers, (ii) characterizing HR by means of function interpolation using (*m, s*)-splines, and (iii) using a k-NN classifier. Interpolation approach provides the best results (around 80% success rate), whereas SVM achieves at most 66% success rate and k-NN was under 70%. Section 6 discusses this issue in depth.

Numerical approximation techniques based on splines are extensively applied in signal processing and surface fitting areas [41–43]. Among the different spline techniques, we selected (*m, s*)-splines [44] for their favorable characteristics to our research: they allow to face multi-variable problems, the problem domain is not required to be a mesh grid (that is, data used to compute interpolation can be at any point in space), and the computational load is low. This technique has not been applied to ambient intelligence system or to classification problems, as far as the author know. However, for our specific case study, results outperform other methodologies.

### 5.1. Problem characterization

**Figure 6.**
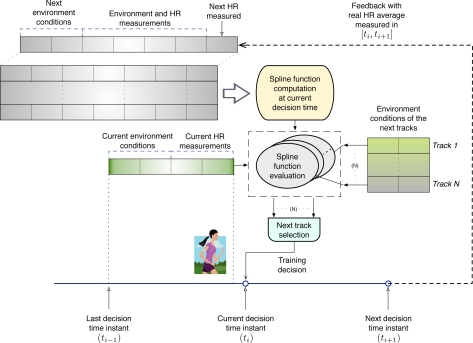
Decision engine operation.

Figure 6 depicts the decision process described in the next sections.

In our problem we assume that the target HR range depends on the desired activity level [45,46]:

- *Moderate activity*. The athlete heart rate is between 50% and 60% of his maximum value.
- *Weight control activity*. The athlete heart rate is between 60% and 70% of his maximum value. item *Cardio training activity*. The athlete heart rate is between 70% and 80% of his maximum value.
- *Anaerobic activity*. The athlete heart rate is between 80% and 90% of his maximum value.
- *Maximum consumed oxygen volume activity*. The athlete heart rate is between 90% and 100% of his maximum value.

where the classical baseline of the HR is considered [5]:

(6) Maximum HR=220−athlete's age

The following input variables are considered:

- Current track environmental temperature, which is considered to be divided in two ranges: “hot” (1) if average temperature in the track is over 25 Celsius degrees or “cold” (0) for lower ones.
- Next track temperature: Hot (1) or cold (0).
- Current track hardness: Difficult (1) or Easy (0) tracks.
- Next track hardness: Difficult (1) or Easy (0).
- Average heart rate during the current track (in beats per minute, bpm).
- Variance of the heart rate during the current track (in bpm^2^).

For each user, a database contains a knowledge base (represented as the input table to the “Spline function computation” box in Figure 6) with recorded sets of these variables and the associated HR value) measured in the experiments. These values are used to construct an interpolation function, which is evaluated for the current conditions, and returns the expected HR. Notice that the decision engine behaves as a learning machine. This is possible because new sensed data (HR) is available at the end of a track (e.g., at *t*_*i*+1_). Then, captured values are incorporated in the data-set, and can be used to re-compute a new spline function. These feedback is represented with a dashed line in Figure 6. The feedback improvement is evaluated in the Section 6.

For every possible next track, based on the input parameters (hardness, temperature, *etc*.) the CN estimates the expected HR (“Spline evaluation box” in Figure 6) using the technique of (*m, s*)-splines described in appendix A. Then, the CN selects the appropriate tracks with the following rule:

- If the expected HR is in the target interval, the track is suitable.
- Otherwise, the track is classified as not suitable.

If there are several suitable tracks, the choice is random. If none is valid, the system select the track with minimal difference between the expected HR and the mean value of the target set.

### 5.2. Example of the decision engine operation

As soon as the CN detects a UE in the IN before a track fork, it executes the previous algorithm. At these decisions instants, the CN executes the algorithm described in the previous section. In this section a detailed example of the operation is provided. The knowledge base is represented in the next table (which contains real values obtained during validation of the case study):

Let us assume that a new track must be selected, that user is 35 years old and its intended activity is “Cardio training”. Then, the target HR is in the interval (129.5, 148) bpm. In addition, let us suppose that input data corresponds to values given in Table 3. That is, there are two possible options, one difficult and one easy track, both with cold temperature. Obviously, the current HR parameters of the athlete are the same. Then, the interpolation function is computed (see Appendix A). In this example, we set *m* = 3 and *s* = 1/2. The result is:

(7) $$\begin{array}{c} 148.36-82.38x_{1}-82.383x_{1}^{2}<<73>>+0.085({\left|0+x_{1}\right|}^{2}+{\left|x_{2}-1\right|}^{2}\\ {+{\left|x_{3}-1\right|}^{2}+{\left|x_{4}-1\right|}^{2}+{\left|x_{5}-154.556\right|}^{2}+{\left|x_{6}-12.414\right|}^{2})}^{1/2}\\ -0.0135{\left({\left|x_{1}+0\right|}^{2}+{\left|x_{2}-1\right|}^{2}+{\left|x_{3}+0\right|}^{2}+{\left|x_{4}-1\right|}^{2}+{\left|x_{5}-137.451\right|}^{2}+\left|x_{6}-11.887\right|\right)}^{1/2} \end{array}$$

where the << 73 >> represents a short form expression of 73 remainder terms.

Next, the two input data sets are used to evaluate the computed interpolation function. The results for the expected HR are HR = 137.3 and HR = 121.9, respectively. The first track is classified as “Suitable”, and the second one as “Unsuitable”. The first track is selected and the CN sends a command to the UE with the instruction of select this track.

## 6. Validation Experiment: Cross-Country Training Circuit

**Figure 7.**
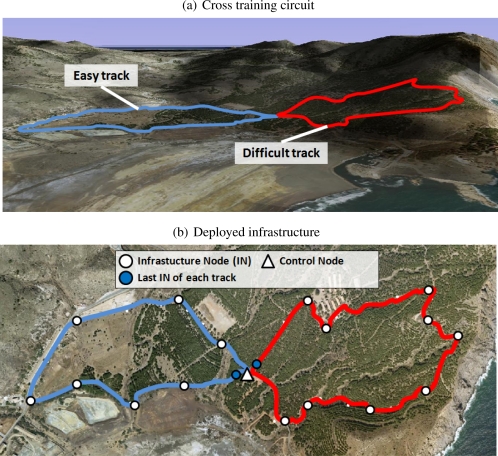
Aerial sights of the training circuit.

The prototype has been validated by means of a cross-country training experiment. The selected area is located near Cartagena (Spain), and it is shown in Figure 7. It consists of two interconnected loops (red and blue) with different hardness and environmental conditions due to:

- Closeness to the sea in some areas of the circuit. For example, training by the coast is characterized by constant winds.
- Different terrain slopes, since part of the circuit is on a hill whose height is 97 m over sea level, with some slopes of 14%.
- Shadow, depending on training hours and the trees along the circuit (as can be observed in the red circuit in Figure 7a). It has influence on temperature.
- Different lengths: 1.1 and 0.9 km for the red and blue tracks, respectively.

The *red* track is considered *difficult* due to of its length and more pronounced slope. The blue track is tagged as *easy*. We deployed ten INs in the difficult sector of the training area and eight in the easy one, as shown in Figure 7b. The CN is placed in the junction of both loops. The network was configured with the protocol parameters that are summarized in Table 1.

In the deployment, communication was extensively tested and performs well in all the tests. The ARQ algorithm in the MAC layer cope with retransmission errors (mainly produced when the UEs were in a shadow area), and automatic retransmissions of broadcast packets solves concerns with location protocol. Simultaneous test were carried out with three runners, and protocols operation were again correct. In this case the CN order a general location (all UEs must respond) in location request orders. The test were limited to three users since this is the number of IMOTE2 available, but we expect similar performance with a large number of users. However, additional test should be performed to confirm this hypothesis.

Besides, some parameters were measured in our test, as a reference of the protocol operation: The round trip time of a message from the CN to a particular UE (or vice versa) was less than 0.55 ms per hop for a packet length of 10 bytes including headers (the length of a command packet). The average location delay was 5.73 s. (the procedure may suffer temporary fading of the communications due to shadow areas). And, finally, in the location procedure, an average number of 1.3 nodes detected each UE (note that more than one node can receive replies from the same UE).

### 6.1. Decision engine conformity tests

The tests were performed for two different athletes. For each of them a specific function is computed from his training data (knowledge base), which were obtained during twelve training sessions. Some test results for three training sessions are shown in Figure 5 and in Table 2. The same sessions were repeated by both athletes, and they followed the same rules:

1. There was an initial warm-up (labeled as WU in Figure 5). During it the athlete trains in the easy sector of the circuit. The data from this sector is discarded.
2. Each training session consists of 12 loops in the circuit (after the warm up sector). At each loop the athlete ran either in the hard or in the easy track. The order was the same for all the sessions (Table 4), and it determined the *course profile*. Besides, the ambient temperature was also monitored. The sessions were performed at the same hour in different days.

With the collected data, the classification algorithm of Section 5 was executed. The conformity of the classification method was measured by means of its degree of discrepancy between the decision engine outcome and the following real HR. That is, if after running in the selected track the average HR was not in the target interval, the classification failed. Otherwise, it was correct. The ratio of successful test is measured in our conformity tests. To perform these tests several samples (test points) were taken out from the knowledge base: for the first runner it included a total of 119 HR values with their associated input variable values, and 14 records were randomly chosen. For the second athlete, with 110 records, 12 were randomly chosen. These records represent 11.8% and 10.9% of their respective knowledge bases. Then, the classification method was applied and the success rate was measured. Table 5 summarizes the results of four conformity tests for the two athletes. The results average the ratios of 30 experiments selecting different test points.

The (*m, s*)-spline decision engine outperformed a SVM and a k-NN classifier for the same knowledge base. We performed the SVM experiments using LibSVM [47], with linear and RBF gaussian kernels (A closely related technique to SVMs are artificial neural networks (ANNs) [48]. Given the bad results with SVMs, which are less prone to overfitting, we discarded ANNs as well.). The corresponding parameters (*C* in the first case and *C* and *γ* in the second one) were chosen using cross-validation. Nonlinear SVMs with RBF kernels usually outperform linear SVMs, and this was confirmed after some experiments. However, our best results in that direction were poor, slightly better that a coin toss (classification accuracy of 66% in the best case). We tried diverse problem precoding alternatives, such as considering ambient temperature and track hardness for two of three previous tracks (instead of only one), but SVM performance did not improve. This seems due to the small number of samples for SVM training and the complexity of the problem (the classes do overlap significantly), which is consistent with the effect of athlete subjective sensations about the intensity of the physical activity (Figure 5) and the nonlinear behavior of HR fluctuations (mentioned in Section 5). Additionally, a k-NN classifier was programmed using Matlab. In this case, accuracy was slightly above of 70% for low values of k (k = 2,3). In case of using a large value of k the accuracy decreases. This may be produced because the number of input points that gives a suitable classification (average HR in target range set) is much less than those unsuitable. That is, there are always more “unsuitable” points than “suitable” ones. Therefore, the k-NN is inefficient.

We did not research tailored kernels to modify the feature space for SVMs, or other techniques such as fuzzy logic, given the disappointing performance of SVMs or k-NNs, and the acceptable results we obtained with (*m, s*)-splines interpolation.

#### Feedback and computational load

Tests performed with the decision engine show how an increase in the number of records increases the quality of the decision. The algorithm was tested selecting a random subset of 25% of the whole data set, than adding a second random subset up to 50% of the whole data set, another one up to 75%, and finally with the whole data set. Results for four random test are summarized in Table 6 for athlete-1 (for athlete-2 results are similar). Clearly, the percentage of valid decisions raise with the size of the data-set. Therefore, as decision engine has more records, accuracy in the prediction increases.

In addition, the computing time and memory requirements for the spline computation was measured (in a laptop computer with a 2 GHz Intel Core Duo CPU, and, 1 GB of DDR2 SDRAM). Both parameters show a linearly dependent of the data set size. The values obtained (≤ 0.5s in all tests, and, near 1 MB for every 100 records) allow to compute interpolation function in real time, even, using a low end performance computer or a smart-phone. Besides, evaluation time is less than 1 ms in all cases evaluated.

#### Environmental parameters influence

Finally, conformity tests were carried out without environmental parameters to measure their influence in the decision. Two experiments have been performed. In the firs one, the information of the temperature (current and future one) was discarded in the computation of the spline, as well as in its evaluations. In the second one, both temperature and hardness (current and future one) was removed. Results are summarized in Table 7, and show a clear reduction in the quality of the classification method. In the case of athlete-1 result is disastrous, with success ratios below 50%.

## 7. Conclusions and Future Work

This work demonstrates the feasibility of ambient intelligence technologies applied to outdoor sports practice. The environment, the athlete condition, and the track configuration are taken into account to provide real-time feedback directly to the user. A main outcome is the design of the classification method, which features a success rate that is close to 80%. Indeed, we demonstrate that classification success depends critically on environmental data, and that feedback positively improves results from the decision engine. Besides, both the infrastructure and the user equipment, which were developed with off-the-shelf sensor technology, behaved well for ambient intelligence applications. The results of this work may be extrapolated to other contexts than sports.

As future work, we aim at developing less intrusive UE devices, design testbeds for different sports, and introduce more parameters and outputs to the decision procedure.

## Figures and Tables

**Table 1.** Protocol parameter selection.

Parameter	Value
*T_QUERY_*	13 s
*T_CN_*	60 s
*T*_*IN*_*DATA*__	120 s
*T*_*UE*_*DATA*__	1 s
*N_QUERY_*	3

**Table 2.** Knowledge base data-set example.

Next		Current				Measured
Hardness (*x*_1_)	Temperature (*x*_2_)	Hardness (*x*_3_)	Temperature (*x*_4_)	Average HR (*x*_5_)	Variance (*x*_6_)	Average HR
0	0	0	0	124.06	200.57	134.78
0	0	0	0	131.52	738.09	139.61
1	0	1	0	108.78	323.5	110.69
0	0	0	0	131.29	200.93	125.67
0	0	1	0	127.59	635.41	148.76
0	0	0	0	148.76	183.11	151.86
0	0	0	0	128.55	312.67	123.14
0	0	0	0	153.13	165.6	155.44
⋮					⋮	
0	1	1	1	81.19	455.38	150.49
0	1	0	1	150.49	297.94	150.55
0	1	1	1	90.66	83.74	142.76
0	1	0	1	137.45	11.89	135.62
0	1	1	1	120.3	96.18	146.39
0	1	1	1	154.56	12.41	143.99

**Table 3.** Input data points for the next training choice.

Next		Current			
Hardness (*x*_1_)	Temperature (*x*_2_)	Hardness (*x*_3_)	Temperature (*x*_4_)	Average HR (*x*_5_)	Variance (*x*_6_)
0	0	0	0	140.08	24.36
1	0	0	0	140.08	24.36

**Table 4.** Course profile of a training session.

Loop	1	2	3	4	5	6	7	8	9	10	11	12	13
Hardness	WU	E	E	E	D	E	E	D	D	E	D	D	D

**Table 5.** Conformity test results.

	Number of records	Test points	Ratio of valid decisions			
			Test 1	Test 2	Test 3	Test 4
athlete-1	119	11.80%	76.40%	76.30%	85.40%	72.40%
athlete-2	110	10.90%	75.30%	75.00%	75.60%	83.70%

**Table 6.** Conformity test and algorithm performance results for athlete-1.

	Test 1				Test 2			
Number of records	28	55	83	110	28	55	83	110
Ratio of test points	10.7%	11.0%	10.8%	10.9%	14.3%	10.9%	12.1%	10.9%
Ratio of valid decisions	33.3%	50.0%	55.5%	83.3%	50.0%	83.3%	90.0%	91.7%
(m,s)	(2,3/2)			(5,3/2)	(2,3/2)			(3,1/2)
Memory used in bytes	322,584	472,384	676,240	3,142,544	317,638	472,072	667,968	1,056,224
Computing time in seconds	0.178	0.225	0.318	0.864	0.175	0.232	0.309	0.442

	Test 3				Test 4			
Number of records	28	55	83	110	28	55	83	110
Ratio of test points	10.7%	11.0%	10.8%	10.9%	14.3%	10.9%	12.1%	10.9%
Ratio of valid decisions	33.3%	50.0%	66.7%	83.3%	66.8%	83.3%	88.9%	100.0%
(m,s)	(2,3/2)				(2,3/2)			(3,1/2)
Memory used in bytes	354,208	525,544	771,688	1,082,816	322,584	472,088	676,552	1,056,456
Computing time in seconds	0.205	0.307	0.456	0.661	0.180	0.202	0.301	0.419

**Table 7.** Influence of environment variables on the percentage of valid decisions.

athlete-1						
	Number of records	Test points	Ratio of valid decisions			
			Test 1	Test 2	Test 3	Test 4
With environment vars.	110	10.90%	83.33%	91.67%	83.33%	100.00%
Without temperature	110	10.90%	66.67%	75.00%	66.67%	83.33%
Without environment vars.	110	10.90%	50.00%	41.67%	33.33%	33.33%

athlete-2						
	Number of records	Test points	Ratio of valid decisions			
			Test 1	Test 2	Test 3	Test 4
With environment vars.	119	11.80%	85.71%	85.71%	76.92%	85.71%
Without temperature	119	11.80%	78.57%	78.57%	61.54%	71.43%
Without environment vars.	119	11.80%	78.57%	71.43%	69.23%	64.29%

## Appendix A. (*m, s*)-Degree Splines Interpolation

The theory of (*m, s*)-degree splines derives from the study of the *semi-Hilbert* space of functions, *X^m,s^*, as is described in [44]. Let *d* ∈ ℕ* be the number of dimensions considered (e.g., six in our problem). Given a set of elements *A^d^*∈ℝ*^d^*, and its corresponding values β ∈ ℝ at those locations (e.g., HR in our problem), the goal is to determine a mapping $\sigma_{\varepsilon }^{d}$ as smooth as possible that approximates the observed data. The problem can be formulated as the minimization of the penalized sum of squares:

(8) $$J_{\varepsilon }^{d}(v)={\left|\rho^{d}v-\beta \right|}^{2}+\varepsilon {\left|v\right|}_{m,s}^{2}$$

where ε *>* 0, *v ∈ X^m,s^*, and *ρ^d^* is an operator defined as

(9) $$\rho^{d}v={\left(v(a)\right)}_{a\in A^{d}}$$

Value $\varepsilon {\left|v\right|}_{m,s}^{2}$ represents a smoothness penalty on function *v*. The *semi-norm* |*v*|*_m,s_* is specifically defined in [44,50]. Then, the *approximation spline of degree* (*m, s*) relative to *A^d^, β*, and *ε*, is a function $\sigma_{\varepsilon }^{d}$ such that ∀*_v_* ∈ *X^m,s^*, $J_{\varepsilon }^{d}(\sigma_{\varepsilon }^{d})\leq J_{\varepsilon }^{d}(v)$. Considering *𝒫_m_* as the ring of real polynomials of *d* variables, *x*_1_, *x*_2_, ..., *x_d_*, and degree ≤ *m*, namely

(10) $$\left\{\begin{array}{cc} p\in {𝒫}_{m}, & p(x)=\sum_{\alpha_{1}+\ldots +\alpha_{d}\leq m}x_{1}^{\alpha_{1}}\ldots x_{d}^{\alpha_{d}};x\in {\mathbb{R}}^{d} \end{array}\;\right\}$$

In [44] it is demonstrated that $\sigma_{\varepsilon }^{d}$ is unique and it can be obtained by solving the following system of equations:

(11) $$\{\begin{array}{cc} \varepsilon C*\lambda_{b}+\sum_{a\in A^{d}}\lambda_{a}K_{2m+2s-n}(b-a)+\sum_{j=1}^{M}C_{j}p_{j}(b)=\beta_{b}, & \varepsilon >0\\ \sum_{a\in A^{d}}\lambda_{a}p_{j}(a)=0, & \forall j=1,\ldots ,M \end{array}$$

where {*p_j_*, 1 ≤ *j* ≤ *M*} is a space basis of *𝒫*_*m*−1_, *M* = dim *𝒫*_*m*−1_, and *λ_a_*, *a* ∈ *A^d^*, *λ_b_*, *b ∈ A^d^*, and *C_j_*, 1 ≤ *j* ≤ *M* are coefficients to be determined. The function *K*_2*m*+2*s*−*n*_(*x*) is

(12) $$K_{2m+2s-n}(x)=\{\begin{array}{cc} {\left|x\right|}^{2m+2s-n}, & \text{si}\;2m+2s\neq 2l,l\in \mathbb{N}*\\ {\left|x\right|}^{2m+2s-n}\text{log}\left|x\right|, & \text{si}\;2m+2s=2l,l\in \mathbb{N}* \end{array}$$

The constant *C** has the following expression:

(13) $$C*=\{\begin{array}{ccc} \frac{{\left(2\pi \right)}^{2m}}{C_{1}} & , & \text{si}\;2m+2s\neq 2l,l\in \mathbb{N}*\\ \frac{{\left(2\pi \right)}^{2m}}{C_{2}} & , & \text{si}\;2m+2s=2l,l\in \mathbb{N}* \end{array}$$

with

(14) $$C_{1}=\frac{\pi^{\left(2m+2s-n/2\right)}\Gamma \left(\frac{n}{2}-m-s\right)}{\Gamma (m+s)},\;\;\;C_{2}=\frac{2\pi^{\left(2m+2s-n/2\right)}{\left(-1\right)}^{\left(m+s-n/2+1\right)}}{\Gamma (m+s)\left(m+s-\frac{n}{2}\right)!}$$

Therefore, approximation (*m, s*)-splines has a coefficient matrix whose diagonal is *ε*C*. Besides, selecting *ε* = 0 transforms the approximation function *σ^d^* in an interpolation function (no error is allowed at the known points).
